# Long-term neprilysin gene transfer is associated with reduced levels of intracellular Abeta and behavioral improvement in APP transgenic mice

**DOI:** 10.1186/1471-2202-9-109

**Published:** 2008-11-12

**Authors:** Brian Spencer, Robert A Marr, Edward Rockenstein, Leslie Crews, Anthony Adame, Rewati Potkar, Christina Patrick, Fred H Gage, Inder M Verma, Eliezer Masliah

**Affiliations:** 1 Department of Neurosciences, University of California San Diego, La Jolla, California 92093, USA; 2 Department of Pathology, University of California San Diego, La Jolla, California 92093, USA; 3 Department of Neuroscience, Rosalind Franklin University of Medicine and Sciences, North Chicago, IL 60064, USA; 4 Laboratory of Genetics, The Salk Institute for Biological Studies, La Jolla, California 92037, USA

## Abstract

**Background:**

Proteolytic degradation has emerged as a key pathway involved in controlling levels of the Alzheimer's disease (AD)-associated amyloid-β (Aβ) peptide in the brain. The endopeptidase, neprilysin, has been implicated as a major Aβ degrading enzyme in mice and humans. Previous short and intermediate term studies have shown the potential therapeutic application of neprilysin by delivering this enzyme into the brain of APP transgenic mice using gene transfer with viral vectors. However the effects of long-term neprilysin gene transfer on other aspects of Aβ associated pathology have not been explored yet in APP transgenic mice.

**Results:**

We show that the sustained expression of neprilysin for up to 6 months lowered not only the amyloid plaque load but also reduced the levels of intracellular Aβ immunoreactivity. This was associated with improved behavioral performance in the water maze and ameliorated the dendritic and synaptic pathology in the APP transgenic mice.

**Conclusion:**

These data support the possibility that long-term neprilysin gene therapy improves behavioral and neurodegenerative pathology by reducing intracellular Aβ.

## Background

Alzheimer's disease (AD) is a progressive neurodegenerative disorder affecting the elderly and is the most common form of dementia [1]. Synaptic pathology is an early indicator of neurodegeneration in the brains of patients with AD [2-4] and in amyloid precursor protein (APP) transgenic (tg) mice [5]. Damage to the nerve terminals is strongly correlated with the severity of the cognitive impairment in patients with AD [2-4]. A key mediator of this disease is believed to be the accumulation of amyloid-β (Aβ) peptides, produced by proteolytic processing of the APP, in the central nervous system [6]. Most recent studies indicate that Aβ oligomers rather than fibrils are responsible for the synaptic pathology in AD [7-9] and other lines of investigation also support a role for intraneuronal accumulation of Aβ in neurodegeneration [10-13].

Extracellular and intracellular accumulation of Aβ peptides might result from alterations in the balance between production, aggregation and degradation [14]. Mutations in the APP and presenillin-1 (PS1) genes have been found to be associated with familial/heritable forms of early-onset AD by increasing the production of Aβ peptides, which has greatly added to our understanding of potential mechanisms leading to aberrant anabolism of Aβ [15-19]. However, the mechanisms of catabolism and clearance of Aβ are less well understood. Endopeptidases, which directly degrade Aβ, have emerged as important players in the homeostatic control of this peptide. Neprilysin (NEP or EC 3.4.24.11) is a zinc metalloendopeptidase that can degrade Aβ [20], and has been identified as a critical Aβ degrading enzyme in the brain [21,22]. Several groups have also reported an inverse correlation between the presence of NEP at the protein or RNA levels and vulnerability to amyloid deposition in both mice and humans [23-30].

The genetic linkage of the NEP gene to AD is currently unclear as several groups have reported no association [31,32], while more have identified an association with the disease [33-36]. Furthermore, chemical inhibition of NEP was shown to result in rapid accumulation of Aβ and pathological deposition in rodents [21,37]. Previously we and other groups have shown that the overexpression of NEP in APP tg mice by gene transfer [38-41], transgenesis [42], or induction [43] resulted in a reduction of Aβ accumulation, neurodegeneration and behavioral deficits. Moreover, the upregulation of NEP by somatostatin or by environmental enrichment has been reported to reduce Aβ levels *in vitro *and *in vivo *[44,45]. Conversely, reduced NEP expression results in amyloid plaque deposition and amyloid angiopathy [46] as well as accumulation of Aβ in the synapses and behavioural deficits [47].

These findings outline the potential application of NEP augmentation to treat AD. However, most previous gene transfer studies with NEP have been for short or intermediate periods of time [38-40,48,49] and the effects of long-term NEP gene transfer on other aspects of Aβ-associated pathology need to be explored in APP tg mice. Among them, recent evidence suggests that accumulation of intracellular Aβ might also play a role in neurodegeneration in transgenic models and in AD [10-13]. We show that the sustained expression of NEP with a lentiviral vector (LV) for up to 6 months, in addition to lowering the amyloid plaque load, reduced the levels of intracellular Aβ immunoreactivity. This was associated with improved behavioral performance in the water maze and ameliorated the dendritic and synaptic pathology in the APP tg mice. These data support the possibility that long-term NEP gene therapy improves behavioral and neurodegenerative pathology by reducing intracellular Aβ.

## Results

### Long-term neprilysin gene transfer reduces the accumulation of intracellular Aβ

**Figure 1:**
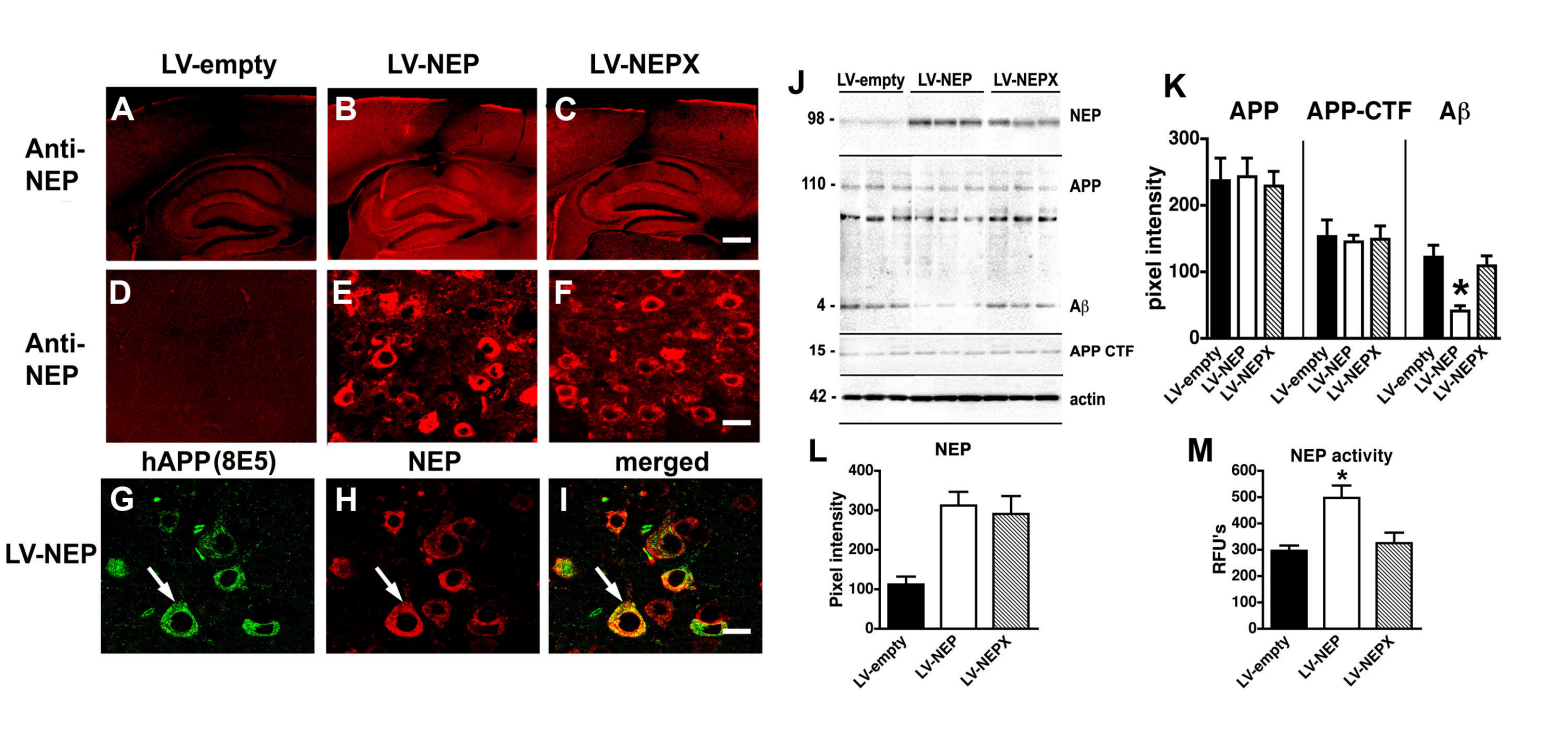
**Immunocytochemical analysis of the levels of NEP expression in brains of APP tg mice that received lentiviral injection**. Panels A-F represent images obtained by LSCM with sections from APP tg mice (12 m/o) injected into the hippocampus and neocortex. (A) Baseline levels of endogenous NEP expression. (B) Increased expression of NEP in the neocortex and hippocampus of mice injected with LV-NEP. (C) Animals that received injections with LV-NEPX expressed levels of NEP comparable to animals injected with LV-NEP. (D-F) Higher magnification views of neurons in the neocortex showing the increased expression of NEP in LV-NEP injected animals. (G-I) Double-labelling confocal analysis demonstrating colocalization of NEP (red channel) in neurons expressing human APP (green channel) in the APP tg mice injected with LV-NEP. Bar is (A-C) 200 μm, (D-F) 10 μm, (G-I) 7 μm. (J-L) Western blot and image analysis of levels of NEP and APP/Aβ (6E10 and CT15) immunoreactivity in APP tg mice treated with lentiviruses. (M) Levels of NEP activity in the cortex of APP tg mice treated with lentiviruses. *p < 0.05 compared to LV-empty by one-way ANOVA with post-hoc Dunnett's, n = 8 mice per group

Our previous study has shown that NEP gene transfer reduces synaptic damage [38]; however, the long-term effects of NEP in other aspects of Aβ pathology and neurodegeneration are less well known. First, to verify that NEP was expressed for the term of the study, immunocytochemical analysis was performed. This study confirmed that in mice that received bilateral injections with LV-NEP at 6 months of age, high levels of NEP expression from the lentiviral vector were detected after 6 months (12 months of age) in the frontal cortex and hippocampus (Fig. 1A–F). Double labelling studies confirmed that in APP tg mice that received LV-NEP injections, the neuronal cells over-expressing APP also expressed NEP from the lentivirus (Fig. 1G–I). The immunoblot analysis confirmed higher levels of NEP immunoreativity in the mice that received the LV-NEP injections compared to LV-empty vector control (Fig. 1J,K). The expression of NEP in the mice injected with LV-NEP resulted in reduced Aβ levels but no effects were observed in full length APP or C-terminal fragments (Fig. 1J,L). The increased levels of NEP immunoreactivity (Fig. 1K) in the LV-NEP injected mice were associated with increased NEP activity compared to the LV-empty vector control and the LV-NEPX (Fig. 1M). The difference in the increase of Nep protein (3 fold) and the increase in Nep activity (1.5 fold) probably represents the kinetics of the enzyme activity of neprilysin cleavage of Aβ.

**Figure 2:**
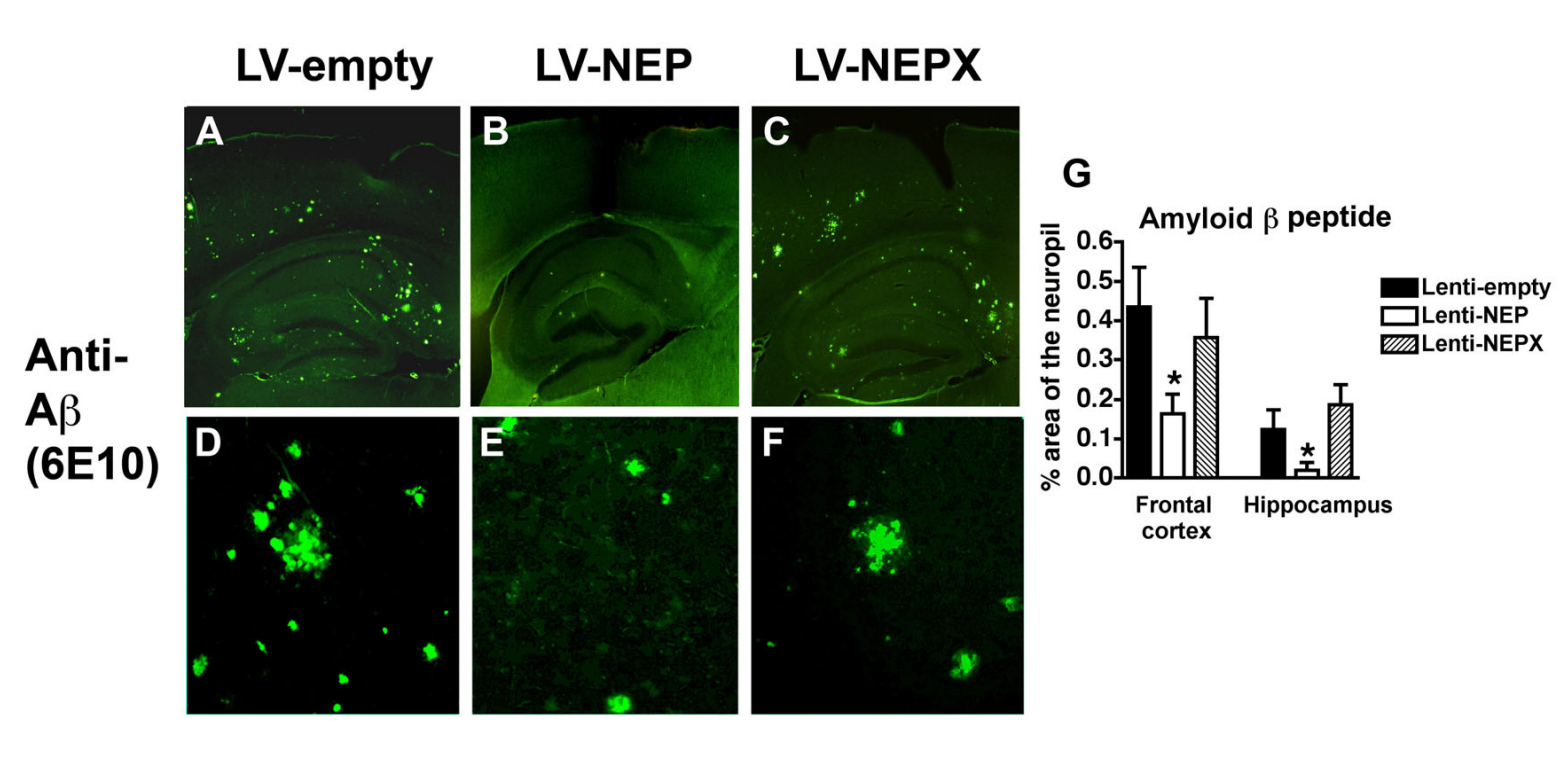
**Neuropathological analysis of Aβ deposit immunoreactivity in the brains of APP tg mice treated with LV-NEP**. (A-C) Low power view (20×) images of extracellular amyloid deposits obtained in the neocortex by LSCM with sections immunostained with an antibody against Aβ (6E10) from the brains of APP tg mice (12 m/o) injected with LV-empty (A), LV-NEP (B) and LV-NEPX (C). (D-F) Higher power view of the amyloid deposits (600×). (G) Image analysis of the percent area of the neuropil occupied by Aβ-immunoreactive deposits shows a significant decrease in mice treated with LV-NEP. *p < 0.05 compared to LV-empty by one-way ANOVA with post-hoc Dunnett's, n = 8 mice per group.

**Figure 3:**
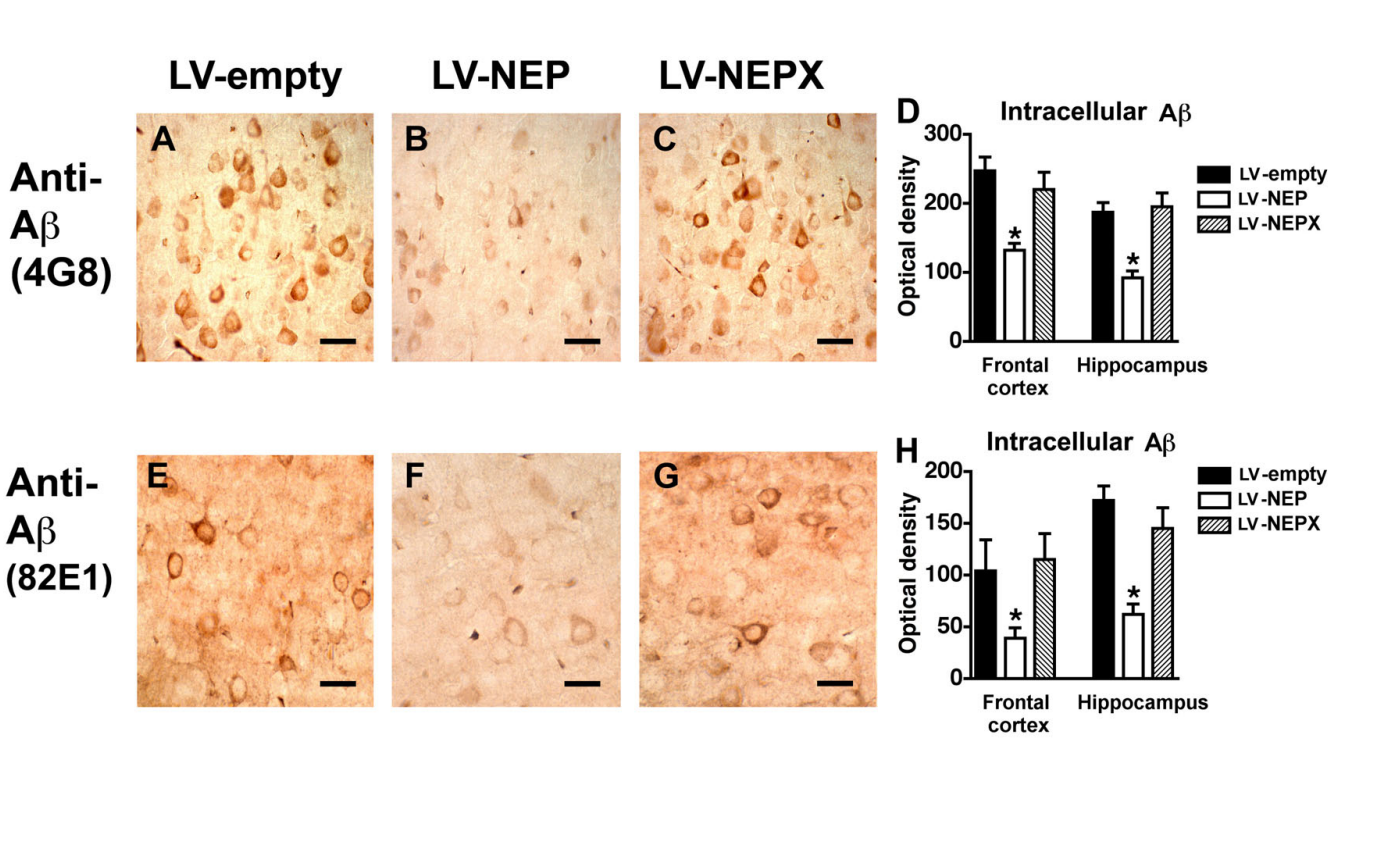
**Neuropathological analysis of intracellular Aβ immunoreactivity in the brains of APP tg mice treated with LV-NEP**. (A-C) Images of intracellular Aβ immunoreactivity obtained in the neocortex with the sections immunostained with an antibody against Aβ (4G8) from the brains of APP tg mice (12 m/o) injected with LV-empty (A), LV-NEP (B) and LV-NEPX (C). (D) Image analysis of the levels of intracellular Aβ-immunoreactivity shows a significant decrease in mice treated with LV-NEP. (E-G) Images of intracellular Aβ immunoreactivity obtained in the neocortex with the sections immunostained with an antibody against Aβ (82E1). (H) Image analysis of the levels of intracellular Aβ-immunoreactivity. *p < 0.05 compared to LV-empty or LV-NEPX by one-way ANOVA with post-hoc Dunnett's, n = 8 mice per group. Bar is (A-C) 20 μm, (E-G) 10 μm.

**Figure 4:**
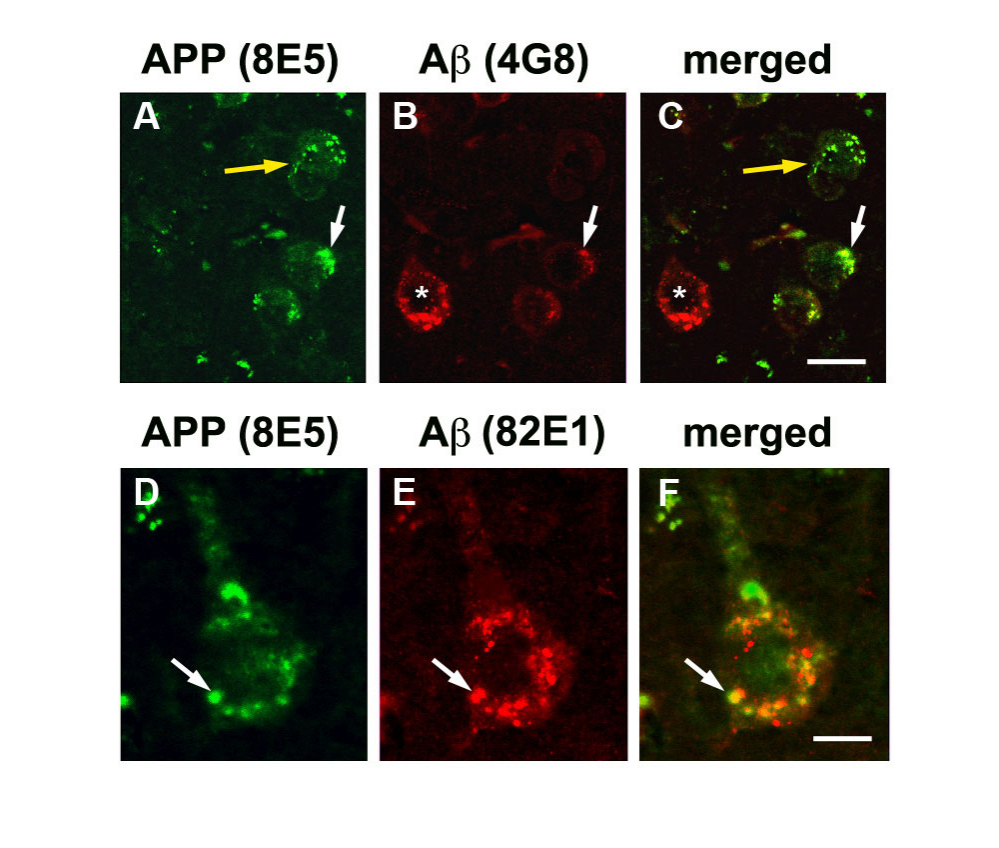
**Double immunolabelling analysis of intracellular Aβ immunoreactivity in the brains of APP tg mice**. (A-C) Images of intracellular APP and Aβ immunoreactivity obtained in the neocortex with sections immunostained with an antibody against Aβ (4G8) (red) and APP (8E5)(green) from the brains of APP tg mice (12 m/o). (D-F) Images of intracellular APP and Aβ immunoreactivity in sections immunostained with an antibody against Aβ (82E1) (red) and APP (8E5)(green). Co-localization of the Aβ and APP staining is identified with a white arrow. Cells staining for APP alone are denoted with a yellow arrow, and cells staining for Aβ alone are denoted with a asterisk. Bar is (A-C) 10 μm, (D-F) 5 μm.

Neuropathological analysis showed that long-term treatment with the LV-NEP reduced levels of amyloid deposition by 48% in the neocortex and 25% in the hippocampus, compared to APP tg mice treated with LV-empty or LV-NEPX (Fig. 2A–G). Although reduced amyloid load is a good indicator of the effects of NEP in this model, amyloid plaques are poorly correlated with cognitive deficits [50,51]. Since recent studies suggest that in addition to the oligomers, accumulation of intracellular Aβ might contribute to neurodegeneration [10-13], we analyzed the levels of neuronal Aβ immunoreactivity with the 4G8 antibody. Image analysis demonstrated that compared to LV-empty and LV-NEPX, the LV-NEP had a profound effect in reducing intracellular Aβ immunoreactivity in pyramidal neurons in the neocortex and hippocampus (Fig. 3A–D). An antibody against the N-terminus of Aβ (clone 82E1) showed similar effects of the LV-NEP reducing levels of intracellular Aβ (Fig. 3E–H). To differentiate the intracellular APP from the Aβ immunoreactive granules, double-labelling studies in the brains of the APP tg mice were performed. About 45% of the neurons that displayed human APP (8E5) contained abundant Aβ immunoreactive (4G8) granules, that in some cases co-localized (Fig. 4A–C). However, there were also neuronal cell bodies with abundant Aβ immunoreactive granules and few or no human APP immunoreactive granules (Fig. 4A–C). This may reflect differences in cellular expression of the β-secretase enzyme involved in the production of Aβ. Similarly results were obtained with the antibody against the N-terminus of Aβ (82E1) and hAPP (8E5) (Fig. 4D–F). Thus the reduction in 4G8 immunostaining we observed in the LV-NEP treated APP tg mice correlates with a reduction in intracellular Aβ.

### Long-term treatment with LV-NEP ameliorates water maze deficits in APP tg mice

**Figure 5:**
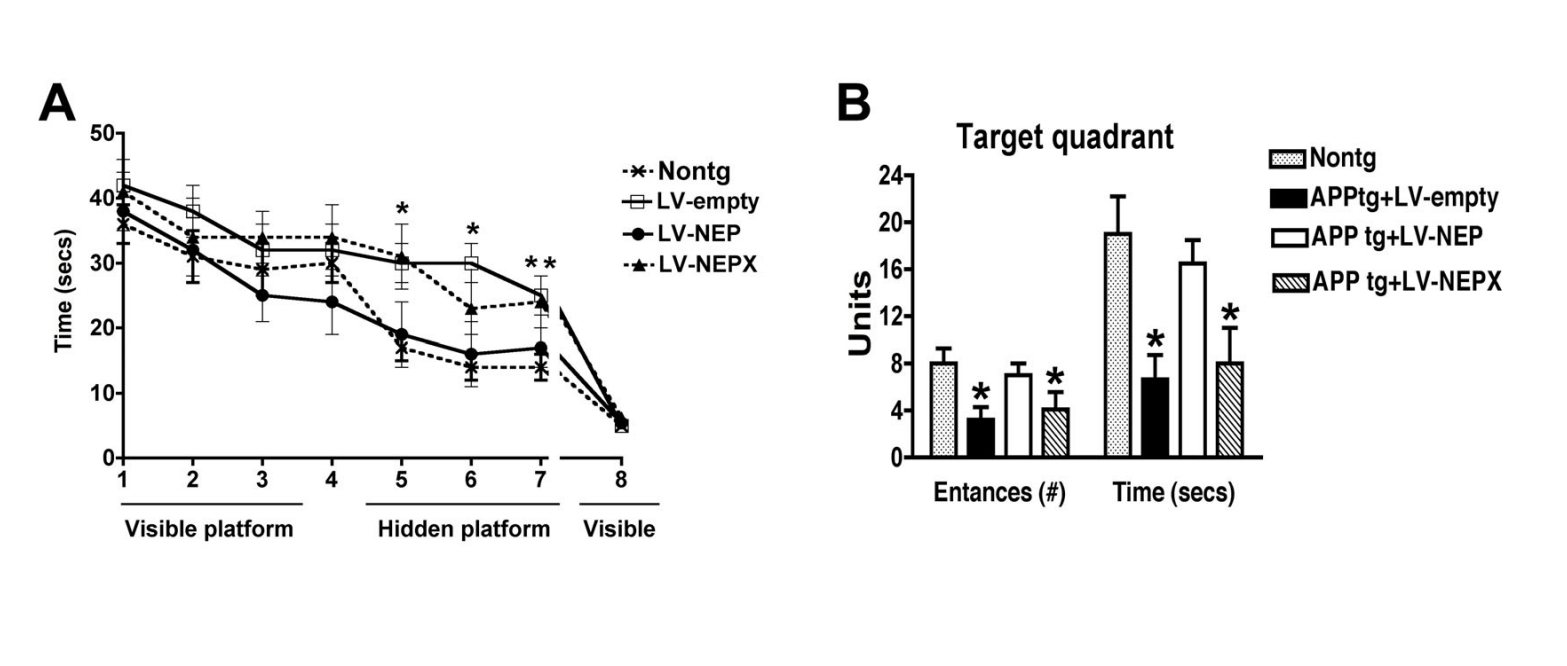
**Behavioral performance in the water maze of APP tg mice that received bilateral injections of LV-NEP into the neocortex and hippocampus**. (A) Test with the visible and submerged platform. *Significant difference on individual days between LV-NEP treated APP tg mice and LV-NEPX or LV-empty treated APP tg mice. **Significant difference for the sum/average of days 4–7 between LV-NEP treated APP tg mice and LV-NEPX or LV-empty treated APP tg mice (p < 0.05 two way ANOVA). (B) Probe test. Mice treated with LV-NEP showed improved behavioral performance compared to LV-empty and LV-NEPX. * = p < 0.05 one way ANOVA when compared to LV-NEP treated APP tg mice or Nontg mice (n = 8 mice per group, F = 4.987, DF = 3, p = 0.0041).

APP tg mice received bilateral injections into the frontal cortex and hippocampus with LV-NEP, or controls (LV-NEPX and LV-empty) and were analyzed in the water maze 6 months later. During the training part of the test when the platform is visible, mice from all 3 groups performed at comparable levels (Fig. 5A). Consistent with results from previous studies [52], at this age APP tg mice injected with LV-empty or the inactive LV-NEPX construct displayed performance deficits in the spatial learning part of the test compared to non-tg controls when the platform was hidden (Fig. 5A). In contrast, mice that received LV-NEP showed improved performance (Fig. 5A). At the end of the test when the platform was visible again, mice from all groups performed similarly (Fig. 5A). Similarly in the probe test, when the platform was removed from the pool, mice that received LV-NEP stayed in the target area for a longer time compared to APP tg mice injected with LV-NEPX or LV-empty (Fig. 5B).

### The effects of LV-NEP on water maze performance and neurodegeneration are associated with reduced intracellular Aβ accumulation

**Figure 6:**
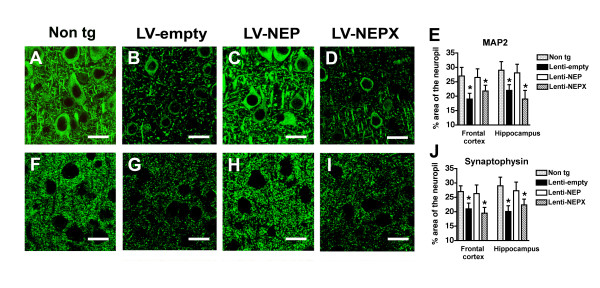
**Effects of LV-NEP treatment on levels of neurodegeneration in APP tg mice**. (A-D) LSCM images of MAP2-immunoreactive dendrites in the neocortex in sections from the brains of a non-tg mouse (12 m/o) (A) or APP tg mice (12 m/o) injected with LV-empty (B), LV-NEP (C) and LV-NEPX (D). (E) Image analysis of the percent area of the neuropil occupied by MAP2 immunoreactive dendrites shows a significant increase in mice treated with LV-NEP. (F-I) LSCM images of synaptophysin-immunoreactive terminals in the neocortex in sections from the brains of a non-tg mouse (12 m/o) (F) or APP tg mice (12 m/o) injected with LV-empty (G), LV-NEP (H) and LV-NEPX (I). (J) Image analysis of the percent area of the neuropil occupied by synaptophysin-immunoreactive terminals shows a significant increase in mice treated with LV-NEP. *p < 0.05 compared to LV-empty by one-way ANOVA with post-hoc Dunnett's. Bar is 15 μm

**Figure 7:**
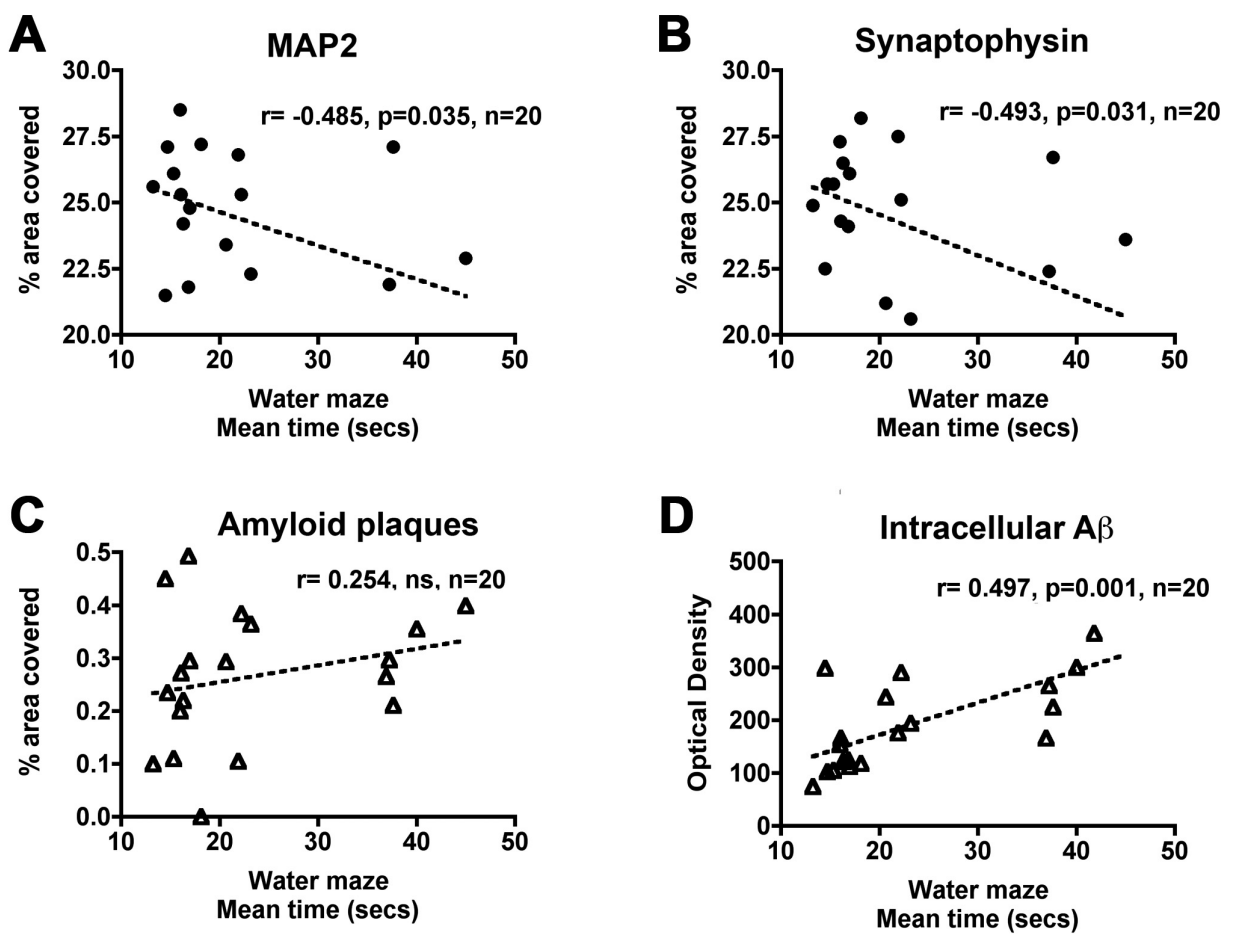
**Linear regression analysis between markers of neurodegeneration or amyloid accumulation and behavioral performance**. (A, B) The average water maze performance in the hidden platform was correlated with the percent area of the neuropil covered by MAP2-immunoreactive dendrites and synaptophysin-immunoreactive terminals. (C, D) While amyloid plaques were not significantly correlated with water maze performance, levels of intracellular Aβ immunoreactivity were significantly correlated.

Previous studies have shown that this line of APP tg mice undergo a progressive loss of dendrites (MAP2) and pre-synaptic (synaptophysin) nerve terminals over time [52,53]. Therefore, we investigated the possibility that long-term treatment with LV-NEP might ameliorate this neurodegenerative pathology. In APP tg mice injected with LV-NEP, MAP2 immunoreactivity was 21% higher in the neocortex and 28% in the hippocampus compared to APP tg mice treated with empty LV-vector or LV-NEPX (p < 0.01) (Fig. 6A–E). Synaptophysin immunoreactivity was increased by 18% in the neocortex and 21% in hippocampus of mice infected with LV-NEP as compared to APP tg mice treated with empty LV-vector or LV-NEPX (p < 0.05) (Fig. 6F–J). This improved density of immunoreactivity for MAP2 and synaptophysin was similar to age matched non-tg mice (Fig 6A, F). Moreover, increased levels of MAP2 and synaptophysin immunoreactivity correlated with the performance in the water maze (Fig. 7A, B). The levels of amyloid load were poorly correlated with the average performance in the hidden platform phase; in contrast, improved performance in the water maze was significantly correlated with reduced levels of intracellular Aβ immunoreactivity in pyramidal neurons (Fig. 7C, D).

## Discussion

The present study showed that long-term gene therapy with LV-NEP for up to 6 months improved behavioral performance and ameliorated the dendritic and synaptic pathology in APP tg mice by reducing the levels of intracellular Aβ immunoreactivity. Previous studies have been focused at analyzing the short-term effects (1 month) of NEP in behavioral deficits and amyloid accumulation in APP tg mice. These studies have shown that delivery of NEP into the hippocampus of APP tg mice by LV, adenoviral-associated viral vectors (AAV) or herpes simplex viral (HSV) vectors can reduce numbers of plaques as well as overall Aβ levels in the CNS [38,39,48,49]. AAV delivered Nep showed localization of the protein at the presynaptic sites whereas with the lentivirus we observed localization to the cell soma and the ER. The reason for this difference in cellular localization is not clear, however it may have to do with the presence of the WPRE translation signal present in the lentivirus vector we used that was not used in the AAV experiments [39]. Similar protein localization differences have been observed for GFP when comparing these two vectors [14].

The vector-delivered NEP resulted in behavioral improvements in the mice. In addition to these studies, recent studies where LV-NEP was delivered into the hippocampus of 3-month old mice showed a reduction in plaques and overall Aβ levels in the CNS as well as improvements in behavior after 4 months [54]. The latter study was performed in mPrP-APP695 mutant mice crossed to presenilin 1 mutant mice [55]. These mice develop plaques and accumulate Aβ at a much greater rate than previous mouse models utilized in the field. For the present study we utilized a more clinically relevant APP tg mouse model [5]. Although mice do not develop the full spectrum of AD, they mimic the human disease in that they develop plaques and display Aβ accumulation and age-dependent synaptic pathology [5,56] and behavioral deficits at a slower rate compared to a recent study [54].

The effects of neprilysin gene therapy might be reflected in a number of compartments, not only in the extracellular Aβ. Although the intracellular pool might represent a small proportion of the total Aβ it might be important in terms of mediating neuronal cell damage [10-13]. The reduction of intracellular Aβ could also be related to a reduction of the extracellular Aβ, with less being endocytosed. In contrast to previous studies that focused on the numbers of plaques and total accumulated levels of Aβ [38,39,48,49,54], we focused our study on the levels of intracellular Aβ. While some studies have suggested that post-transcriptionally-modified Aβ fragments [57] and oligomers [7-9] might be responsible for the neurotoxic effects, others have suggested that accumulation of intracellular Aβ might also play a role [10-13]. This is a concern because recent evidence suggests that accumulation of intracellular Aβ may play a role in neuronal dysfunction and degeneration by disturbing ERK-CREB signalling and increasing intracellular calcium levels [12,58].

We found that levels of intracellular Aβ were reduced in areas where the LV-NEP was injected. Moreover, the reduction in intracellular Aβ immunoreactivity correlated with the improved behavioral performance in the mice. This is consistent with a study in Drosophila that showed that overexpression of NEP reduces the pathology associated with tg expression of intracellular Aβ_1–42 _[59]. The mechanisms through which NEP might reduce intracellular Aβ are not clear, however NEP is a membrane-bound enzyme that is distributed both in the plasma membrane and endo-membrane system [60]. Thus, interactions might occur at the endoplasmic reticulum where previous studies have shown that intracellular Aβ accumulates [10-13].

It has become increasingly clear in recent years that NEP may be an important player in the treatment of AD. Several genetic linkage studies have now reported an association between NEP polymorphisms and risk for AD [33-36]. Furthermore, a potential mechanism has been identified by which NEP down-regulation with age can increase vulnerability to amyloid pathology via reduced levels of somatostatin [44]. In addition, NEP levels and activity are significantly reduced in the brains of AD patients [30,61-63]. Therefore, replacement therapy might require long-term NEP delivery, which might raise concerns about possible toxic effects. For this reason, we extended our studies to a 6-month duration of therapy. It is important to emphasize that compared to others, in our study injections were placed both in the hippocampus and neocortex and we also analyzed the integrity of the neuronal structure with antibodies against synaptophysin and MAP2.

## Conclusion

The present study confirmed that long-term LV-NEP ameliorated the neurodegenerative pathology in APP tg mice that was associated with improved water maze performance. It is worth noting that the local levels of the LV-driven transgene expression are relatively low and it is unclear if much higher levels of expression for a long time might have undesired side effects. Future studies will be needed to address this issue.

In conclusion, these data support the possibility that long-term NEP gene therapy improves behavioral and neurodegenerative pathology by reducing intracellular Aβ and support the potential for NEP augmentation in the treatment of AD.

## Methods

### Preparation of lentiviral vectors

Design of LV constructs was described previously [38]. We produced LV expressing NEP or NEPX (an inactive version, bearing a point mutation E585V) under the control of the CMV promoter, or empty vector (LV-empty) using a four-plasmid transfection system, as previously described [64-66]. Briefly, 293T cells were transfected with vector and packaging plasmids, the supernatants collected and vectors concentrated by centrifugation. The LV titers were estimated by measuring the amount of HIV p24 gag antigen by ELISA (Perkin Elmer Life Science, Boston, MA) (100,000 TU/ng p24) or by flow cytometry using a NEP-specific antibody (56C6, Research Diagnostics, Inc., Concord, MA).

### Transgenic mouse generation and intracerebral injections of lentiviral vectors

For this study we used tg mice expressing the mutant human amyloid precursor protein (hAPP^V717F/K670M/N671L^) under the control of the platelet derived growth factor promoter (PDAPP minigene) [5]. This line (J9M) of APP tg mice was selected because these mice express moderate levels of APP/Aβ and display neurodegenerative pathology and behavioral deficits associated with Aβ_1–42 _production [5]. For this purpose, 6-month old APP tg mice (n = 24) received bilateral injections with 2 μl of the LV preparations (1.5 × 10^7 ^TU) into the frontal cortex and hippocampus (using a 5 μl Hamilton syringe, 0.25 μl/min). Mice received bilateral injections with LV-NEP (n = 8), LV-NEPX (inactive) (n = 8), or empty LV vector (LV-empty) alone (n = 8). Six months post-injection (12 months old) mice were tested in the water maze.

### Water maze testing

As previously described [67], in order to evaluate the functional effects of long LV-NEP treatment in mice, groups of APP tg animals were tested in the water maze. For this purpose, a pool (diameter 180 cm) was filled with opaque water (24°C) and mice were first trained to locate a visible platform (days 1–3) and then a submerged hidden platform (days 4–7) in three daily trials 2–3 min apart. Mice that failed to find the hidden platform within 90 s were placed on it for 30 s. The same platform location was used for all sessions and all mice. The starting point at which each mouse was placed into the water was changed randomly between two alternative entry points located at a similar distance from the platform. On day 8, another visible platform trial was performed to exclude differences in motivation and fatigue. Time to reach the platform (latency), path length, and swim speed were recorded with a Noldus Instruments EthoVision video tracking system (San Diego Instruments, San Diego, CA) set to analyze two samples per second. All experiments described were approved by the animal subjects committee at the University of California at San Diego (UCSD) and were performed according to NIH recommendations for animal use.

### Tissue processing

Brains were removed and divided sagittaly. One hemibrain was post-fixed in phosphate-buffered 4% paraformaldehyde (pH 7.4) at 4°C for 48 hrs and sectioned at 40 μm with a Vibratome 2000 (Leica, Germany), while the other hemibrain was snap-frozen and stored at -70°C for immunoblot analysis.

### Analysis of APP/Aβ levels by immunoblot

For Western blot analysis, 20 μg per lane of cytosolic and particulate fractions, assayed by the BCA method (Pierce Biotechnology, Rockford, IL), were loaded into 4–12% SDS-PAGE gels and blotted onto polyvinylidene fluoride (PVDF) membranes. Blots were incubated with antibodies against APP/Aβ (mouse monoclonal, clone 6E10, Signet Laboratories, Dedham, MA or rabbit polyclonal, clone CT15, personal gift of E. Koo) and NEP (mouse monoclonal, clone CD10, Abcam, Cambridge, MA) followed by secondary antibodies tagged with horseradish peroxidase (HRP, Santa Cruz Biotechnology, Inc.) and visualized by enhanced chemiluminescence and analyzed with a Versadoc XL imaging apparatus (BioRad, Hercules, CA). Analysis of actin levels was used as a loading control.

### Analysis of NEP activity

The proteolytic activity of NEP was measured as previously described [40] using the substrate 3-dansyl-D-Ala-Gly-p-(nitro)-Phe-Gly (DAGNPG; Sigma). Cell lysate was incubated with 50 μM DAGNPG and 1 μM captopril (to inhibit any ACE cleavage of DAGNPG) in a volume of 200 μl at 37°C. Reactions were stopped by heating samples to 100°C for 5 min, then centrifuging. The supernatant was diluted into 50 mM Tris (pH 7.4) and fluorescence determined using a Victor2 multilabel plate reader (excitation 342 nm; emission 562 nm).

### Analysis of Aβ levels and amyloid plaque load by immunocytochemistry

To evaluate the amyloid plaque load, briefly as previously described[68], vibratome sections were incubated overnight at 4°C with the mouse monoclonal antibody against Aβ (clone 6E10, 1:1000, Signet Laboratories, Dedham, MA) followed by fluorescein isothiocyanate (FITC)-conjugated anti-mouse IgG (Vector Laboratories, Burlingame, CA). The FITC-labelled sections were imaged with the laser scanning confocal microscope (LSCM, MRC1024, BioRad) as described previously [5]. Digitized images were analyzed with the NIH Image 1.43 program to determine the percent area of the neuropil occupied by Aβ-immunoreactive deposits in the frontal cortex and hippocampus. To evaluate effects on intracellular Aβ, sections were immunolabelled with the mouse monoclonal antibody against Aβ (clone 4G8; 1:600, Senetek, Napa, CA) and with the antibody against the N-terminus of Aβ (aa 1–16; clone 82E1; Immunobiological Laboratorie Co. Gunma, Japan) followed by incubation with secondary biotinylated anti-mouse IgG, followed by ABC and DAB. Sections were transferred to SuperFrost slides (Fisher Scientific, Tustin, CA) and mounted under glass coverslips with anti-fading media (Vector Laboratories). All sections were processed under the same standardized conditions. Three immunolabelled sections were analyzed per mouse and the average of individual measurements was used to calculate group means.

### Analysis NEP expression, double immunolabelling and neurodegeneration

To verify the expression levels of NEP, vibratome sections were immunolabelled with a monoclonal antibody against NEP (CD10, 1:10,000, Abcam, Cambridge, MA) detected with the Tyramide Signal Amplification™-Direct (Red) system (1:100, NEN Life Sciences, Boston, MA). To evaluate the co-localization between APP and NEP, or the intracellular distribution of APP and Aβ double immunocytochemical analysis was performed as previously described [69]. For this purpose, vibratome sections were immunolabelled with a monoclonal antibody against NEP (1:10,000, Abcam) or Aβ (clone 4G8; 1:600, Senetek, Napa, CA) detected with the Tyramide Signal Amplification™-Direct (Red) system (1:100, NEN Life Sciences, Boston, MA) and the mouse monoclonal antibodies against human APP (1:500, 8E5 clone, Elan Pharmaceuticals, South San Francisco, CA) detected with FITC-conjugated secondary antibodies (1:75, Vector Laboratories) [69]. All sections were processed simultaneously under the same conditions and experiments were performed twice to assess reproducibility. Sections were imaged with a Zeiss 63× (N.A. 1.4) objective on an Axiovert 35 microscope (Zeiss, Germany) with an attached MRC1024 LSCM system (BioRad) [69]. To confirm the specificity of primary antibodies, control experiments were performed where sections were incubated overnight in the absence of primary antibody (deleted) or preimmune serum and primary antibody alone.

The integrity of the neuronal structure was evaluated as previously described [68,70]; briefly, blind-coded, 40-μm thick vibratome sections from mouse brains fixed in 4% paraformaldehyde were immunolabelled with the mouse monoclonal antibodies against synaptophysin (synaptic marker, 1:20, Chemicon, Temecula, CA) and microtubule associated protein-2 (MAP2, dendritic marker, 1:40, Chemicon). After overnight incubation with the MAP2 and synaptophysin, sections were incubated with FITC-conjugated horse anti-mouse IgG secondary antibody (1:75, Vector Laboratories), transferred to SuperFrost slides (Fisher Scientific) and mounted under glass coverslips with anti-fading media (Vector Laboratories). All sections were processed under the same standardized conditions. The immunolabelled blind-coded sections were serially imaged with the LSCM (MRC1024, BioRad) and analyzed with the Image 1.43 program (NIH), as previously described [71,72]. For each mouse, a total of 3 sections were analyzed and for each section, 4 fields in the frontal cortex and hippocampus were examined. For synaptophysin and MAP2, results were expressed as percent area of the neuropil occupied by immunoreactive terminals and dendrites.

### Statistical analyses

Analyses were carried out with the StatView 5.0 program (SAS Institute Inc., Cary, NC). Differences among means were assessed by one-way ANOVA with post-hoc Dunnett's or Tukey-Kramer tests. All values in the figures are expressed as means ± SEM. Comparisons between 2 groups were done with the unpaired two-tailed Student's t-test. Correlation studies were carried out by simple regression analysis and the null hypothesis was rejected at the 0.05 level. To evaluate the effects of interactions between genotype and treatment 2-way ANOVA was used with the SuperANOVA software package (SAS Institute Inc., Cary, NC).

## Authors' contributions

BS participated in the design of the study, prepared lentiviral vectors, performed *in vitro *experiments and partially drafted the manuscript. RAM participated in the design of the study, prepared lentiviral vectors, performed behavioral analyses and partially drafted the manuscript. ER performed *in vivo *experiments in the transgenic mice. LC performed *in vitro *experiments and edited the manuscript. AA and CP performed immunocytochemical and immunohistochemical experiments. RP performed *in vitro *experiments. FHG and IMV participated in the design of the study. EM participated in the design of the study, performed confocal microscopy and statistical analyses and drafted the manuscript. All authors read and approved the final manuscript.
